# Expression Characteristics and Significant Prognostic Values of PGK1 in Breast Cancer

**DOI:** 10.3389/fmolb.2021.695420

**Published:** 2021-07-05

**Authors:** Yanping Li, Shanshan Wang, Xiaoyuan Zhang, Rui Yang, Xiaonan Wei, Ruirong Yan, Yaru Jiang, Wenzhi Shen

**Affiliations:** ^1^ Department of Pathology and Institute of Precision Medicine, Jining Medical University, Jining, China; ^2^ Institute of Breast Research, Jining Medical University, Jining, China

**Keywords:** PGK1, breast cancer, prognostic, public databases, prognostic value, comprehensive analysis 3

## Abstract

It was proven that PGK1 plays a vital role in the proliferation, migration, and invasion of human breast cancer. However, the correlation of PGK1 mRNA and protein expression with clinicopathologic characteristics and prognostic values according to various kinds of breast cancer patient classifications remains unsufficient. Here, we analyzed data from the Oncomine database, Breast cancer Gene-Expression Miner v4.5, TNMplot, MuTarget, PrognoScan database, and clinical bioinformatics to investigate PGK1 expression distribution and prognostic value in breast cancer patients. Our study revealed that the mRNA and protein expression levels of PGK1 were up-regulated in various clinicopathologic types of breast cancer. Moreover, the expression of PGK1 was correlated with mutations of common tumor suppressor genes TP53 and CDH1. In addition, we found that high mRNA level of PGK1 was significantly associated with poor OS, RFS, and DMFS. Notably, Cox regression analysis showed that PGK1 could be used as an independent prognostic marker. In summary, the aforementioned findings suggested that PGK1 might be not only explored as a potential biomarker, but also combined with TP53/CDH1 for chemotherapy in breast cancer.

## Introduction

Breast cancer is one of the leading diseases afflicting women worldwide. According to the data of International Agency for Research on Cancer (IARC) in 2020 (Siegel et al., 2021; Sung et al., 2021), the annual prevalence of breast cancer increased to 2.26 million cases and 0.68 million relevant death cases globally. Over the last few decades, with the development of the detection methods and treatment strategy, the survival rate of breast cancer has been improved. However, it remains an enormous health burden for woman globally (Qiu et al., 2018). In addition, breast cancer patients with metastases have an extremely poor prognosis (Nakhjavani et al., 2019). The classification and pathogenesis of BRCA is quite complex. It involves multiple processes such as cell cycle disorder and metabolic abnormalities, illustrating the interactions and functions of multiple genes at multiple processes (Saini et al., 2019). Thus, screening of effective biomarker networks is urgently needed for the diagnosis, treatment, and prognostic assessment of BRCA.

Notably, Warburg effect Warburg et al. (1927), Yang et al. (2018) as one of the prominent characteristics of cancer cell, was closely related with cancer progression. The most characteristic feature of tumor cells is that they are mainly powered by the glycolytic pathway even when they are well oxygenated (Warburg, 1956; Han et al., 2019). Phosphoglycerate kinase 1 (PGK1) works as an important rate-limiting enzyme in the metabolic glycolysis pathway which could catalyze the conversion of 1,3-diphosphoglycerate to 3-phosphoglycerate and generate a molecule of ATP (Vas et al., 2010; He et al., 2019). In addition to the important role in glycolysis, PGK1 was also proved to have critical function in tumor progression. It was reported that PGK1 was high expressed and could be used as a diagnostic marker in many tumors including endometrial cancer, lung cancer, colon cancer, and gastric cancer (Ahmad et al., 2013; Tekade and Sun, 2017; Zhang et al., 2018). Other reports also show that PGK1 expression is associated with tumor progression and prognosis of patients with gallbladder cancer (Lay et al., 2000). However, the role and clinical significance of PGK1 in different pathology subtype of breast cancer remain unclear.

In this study, we analyzed data from various online databases and revealed that the mRNA and protein expression levels of PGK1 were up-regulated in various clinicopathologic types of breast cancer. Moreover, the expression of PGK1 was correlated with mutations of common tumor suppressor genes TP53 and CDH1. In addition, we also found that high mRNA level of PGK1 was significantly associated with poor OS, RFS, and DMFS. Notably, Cox regression analysis showed that PGK1 could be used as an independent prognostic marker. In summary, the aforementioned findings suggested that PGK1 might be not only explored as a potential biomarker, but also combined with TP53/CDH1 for chemotherapy in breast cancer.

## Materials and Methods

### Oncomine Database Analysis

The Oncomine database (https: //www. oncomine.org/resource/login.html), was used to determine the transcription expression level of PGK1 gene in breast cancer (Toruner et al., 2004; Viala et al., 2017). The expression levels of PGK1 mRNA (log2-transformed) were assessed in BRCA tissue relative to its expression in normal tissue. To obtain the most significant PGK1 expression, thresholds were set as below: gene rank, 10%; fold change, 2; and *p*-value, 0.01.

### UALCAN Database Analysis

To determine the reliability of the differential expression data, the UALCAN database was selected for further verification. UALCAN is a comprehensive, user-friendly, and interactive web resource for analyzing cancer OMICS data. It was designed to provide easy access to publicly available cancer OMICS data (TCGA and MET500), provide graphs and plots depicting pan-cancer gene expression and patient survival information based on gene expression, evaluate gene expression in molecular subtypes of breast cancer, and evaluate epigenetic regulation of gene expression by promoter methylation and correlate other clinicopathological features (Chandrashekar et al., 2017). UALCAN is publicly available at http://ualcan.path.uab.edu.

### TNMplot Database Analysis

The TNMplot tool (https://www.tnmplot.com/) is suitable for differential gene expression analysis in tumor tissues, normal tissues, and metastatic tissues. TNMplot includes 56,938 unique multilevel quality-controlled samples including Genechip from GEO: 3,691 normal, 29,376 tumor, and 453 metastatic samples and RNA-seq from TCGA: 730 normal, 9,886 tumor, and 394 metastatic samples (Bartha and Győrffy, 2021). The expression of PGK1 in normal, cancerous, and metastatic tissues were compared and analyzed using this tool.

### Differentially Expressed PGK1 at Protein Level

In addition to the Oncomine and UALCAN database, the protein expression analysis of PGK1 was studied using the data from the HPA (http://www.proteinatlas.org). HPA is a platform that contains representative immunohistochemistry-based protein expression data for 20 highly common kinds of cancers (Thul et al., 2017). In this study, immunohistochemistry images of protein expression of PGK1 between normal and BRCA samples were directly visualized by HPA.

### Breast Cancer Gene-Expression Miner v4.5

The expression and prognostic value of PGK1 in breast cancer were assessed by using Breast Cancer Gene-Expression Miner v4.5 online data set (http://bcgenex.centregauducheau.fr). The online dataset is a statistical mining tool of published annotated breast cancer transcriptomic data including DNA microarrays, RNA-seq with large amount of published annotated genomic data and can perform statistical analysis of gene expression, correlation and prognosis. The data on this website were last updated in June 2020 (Jézéquel et al., 2021). The relationship between PGK1 and the clinic pathologic parameters of breast cancer were evaluated by using bc-GenExMiner v4.5.

### PrognoScan Online Database

PrognoScan online database was used to explore the underlying tumor biomarkers or therapeutic targets (http://www.prognoscan.org/) (Mizuno et al., 2009). In this study, the PrognoScan database analysis was performed to validate the prognostic significance of PGK1 mRNA expression in breast cancer patients, and a corrected *p*-value was set to adjust the threshold. According to the median expression of the genes, the online tool could divide the expression of PGK1 into “high” group or “low” group. Blue curves correspond to low PGK1 expression, while red curves to high PGK1 expression.

### MuTarget Analysis

The MuTarget includes two independent analyses platform which links target gene expression changes with common mutation status in human tumors. By using this, the changes of target gene expression associated to a gene mutation and mutations altering the expression of a selected gene could be identified. The R statistical tool was used in all data processing steps. RNA-sequencing and mutation data were acquired from TCGA database. In this established database, 7,876 solid tumor samples from 18 various tumor types with both RNA-seq data and somatic mutation (Nagy and Győrffy, 2021). The utility of this approach is presented *via* three analyses in breast cancer: gene expression changes related to CDH1 mutations, gene expression changes related to TP53 mutations, and mutations mediated altered progesterone receptor (PGR) expression. The breast cancer database was split into equally sized training and test sets, and these data sets were analyzed independently. Based on the Mann-Whitney *p*-value and mean FC, significant genes were selected for the test, where we used the default thresh-olds of *p* ≤ 0.01 and 0.714 > FC > 1.4.

### Construction of Related Gene Networks, GO, and KEGG Pathway Enrichment Analysis

GeneMANIA (http://www.genem ania.org) provides a flexible web interface for deriving hypotheses based on gene functions (Warde-Farley et al., 2010), which generates a list of genes with similar functions to the query gene and constructs an interactive functional-association network to illustrate relationships between genes and datasets. In the present study, PGK1 was submitted to the GeneMANIA to interpret the functional association network among PGK1 and their related genes. Next, we used Web Gestalt (http://www.webgestalt.org/), which is a functional enrichment analysis web tool with continuously updated and effectively reduced data redundancy (Liao et al., 2019). KEGG pathways and GO functions analysis of PGK1 and their 20 associated genes were enriched by the Web Gestalt. The method of interest is selected in Over-Representation Analysis (ORA). The GO functional enrichment was performed in the biological process no Redundant (BP), cellular component no Redundant (CC), and molecular function no Redundant (MF). The pathway analysis was processed in the KEGG pathway.

### Assistant for Clinical Bioinformatics Analysis

Assistant for clinical bioinformatics database (www.aclbi.com) was used to study the influence of genes and clinical factors, such as age, sex, and TNM stages on prognosis. Univariate and multivariate cox regression analysis were performed to identify the proper terms to build the nomogram. Based on the multivariate Cox proportional hazards analysis results, a nomogram was developed to predict the X-year overall recurrence (Lin et al., 2020; Zhang et al., 2020). The nomogram provided a graphical representation of the factors, which can be used to calculate the risk of recurrence for an individual patient by the points associated with each risk factor. All analytical methods above and R packages were performed using R software version v4.0.3 (The R Foundation for Statistical Computing, 2020); *p* < 0.05 was considered as statistically significant.

## Results

### High mRNA Expression of PGK1 was Found in Human Breast Cancer

**FIGURE 1:**
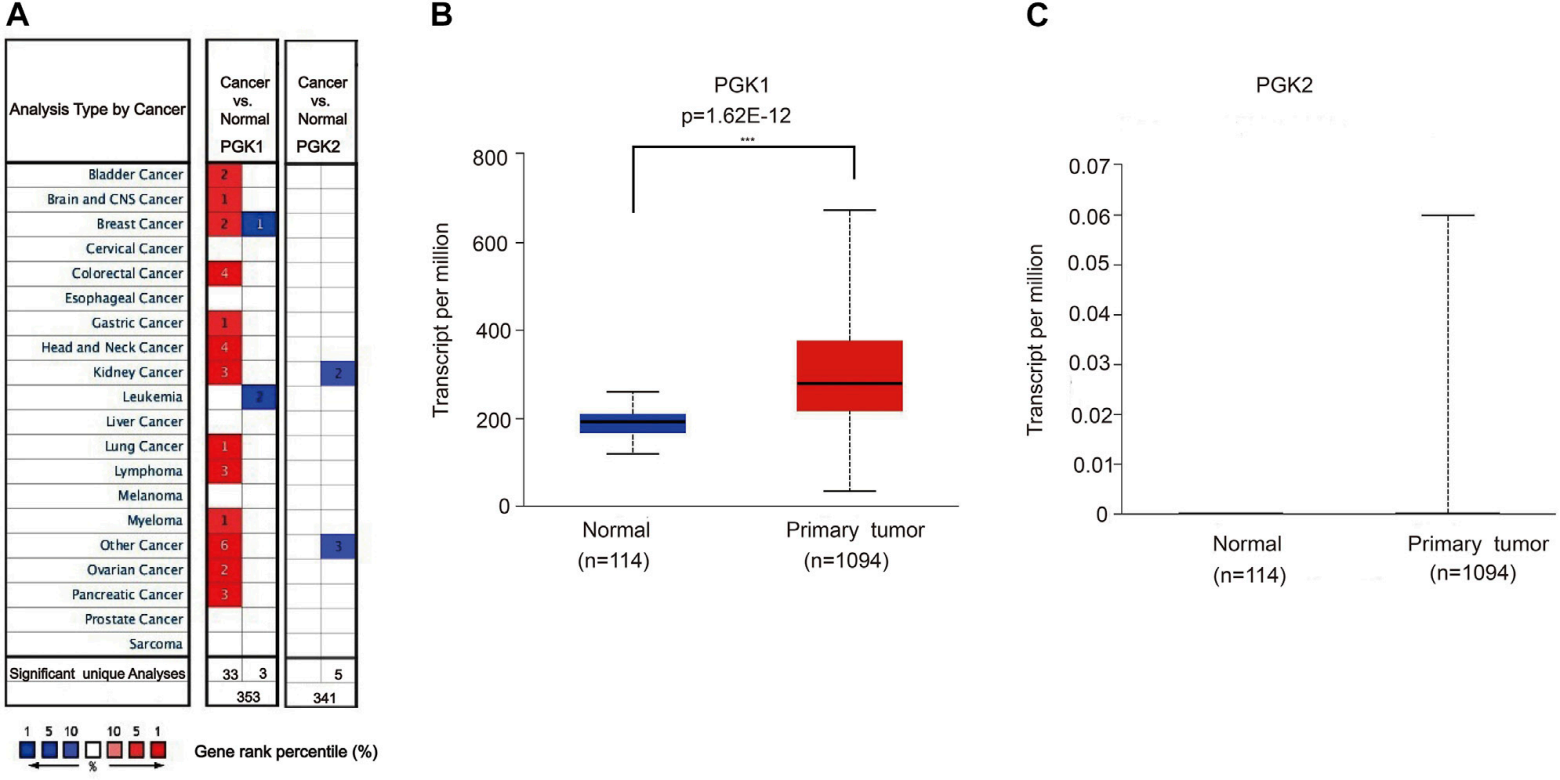
**(A)** Transcriptional expressions of PGK1 and PGK2 in 20 types of cancers (cancer vs. corresponding normal tissue) were evaluated using the Oncomine database. Comparing the data by the *t*-test and cut-off *p*-value and fold change, following: *p*-value < 0.0001, fold change = 2, gene rank = 10%. Red represents significant overexpression, blue represents reduced expression. PGK1, phosphoglycerate kinase 1; PGK2, phosphoglycerate kinase 2. **(B,C)** High mRNA expressions of PGK1and PGK2 in patients with breast cancer and normal breast tissues by UALCAN (TCGA database), *** means *p* < 0.001.

Phosphoglycerate kinase (PGK) is the only kinase that plays an important role in glycolysis. It catalyzes the transfer of high-energy phosphate from the position 1 of 3-bisphosphoglycerate (1, 3-BPG) to ADP, which generates 3-phosphoglycerate (3-PG) and ATP. PGK includes two isoenzymes, phosphoglycerate kinase 1 (PGK1) and phosphoglycerate kinase 2 (PGK2), and the heterogeneity of their amino acid sequences is up to 88%. By using the TCGA and Oncomine database, the expression profile was examined. We determined the expression of PGK1 and PGK2 in various human cancer types and found that it was significantly elevated in breast cancer vs. the normal tissue (Figures 1A,B). We further compared the mRNA expression of PGK1 in tumor samples and normal tissues using the UALCAN database. Analysis of RNA sequence data based on the TCGA database, consistent with the Oncomine data, showed that PGK1 expression was obviously up-regulated in BRCA tissues vs. the normal controls (*p* = 1.62 × 10^−12^) (Figure 1B).

**FIGURE 2:**
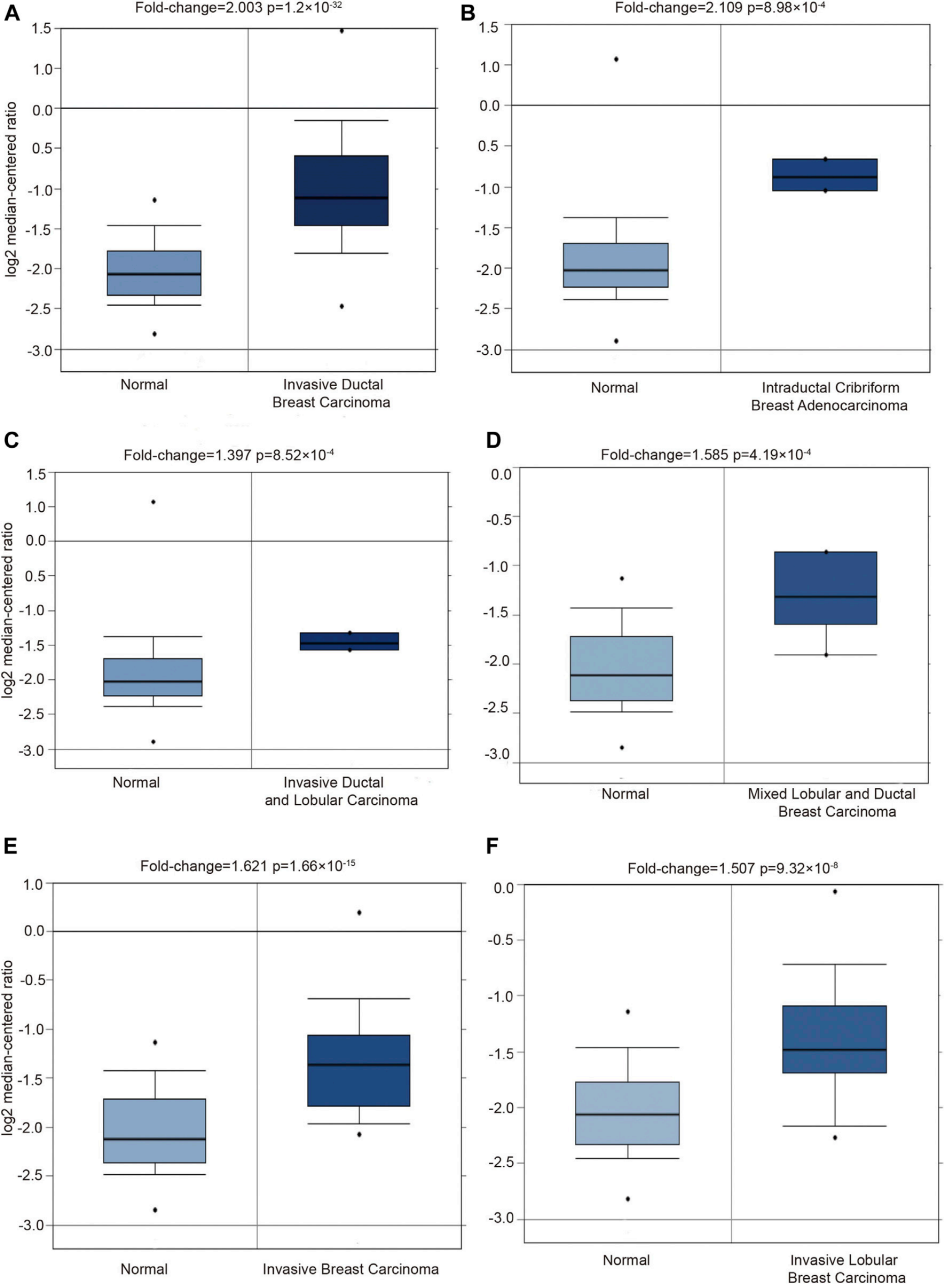
Detecting the PGK1 gene expression in six types breast cancer by using Oncomine database. Six boxplots originated from gene expression data in the Oncomine, comparing the expression of PGK1 in normal tissues and six types of breast cancer tissues as follows: invasive ductal breast carcinoma **(A)**, intraductal cribriform breast adenocarcinoma **(B)**, invasive ductal and lobular carcinoma **(C)**, mixed ductal and lobular breast carcinoma **(D)**, invasive breast carcinoma **(E)** and invasive lobular breast carcinoma **(F)**.

**FIGURE 3:**
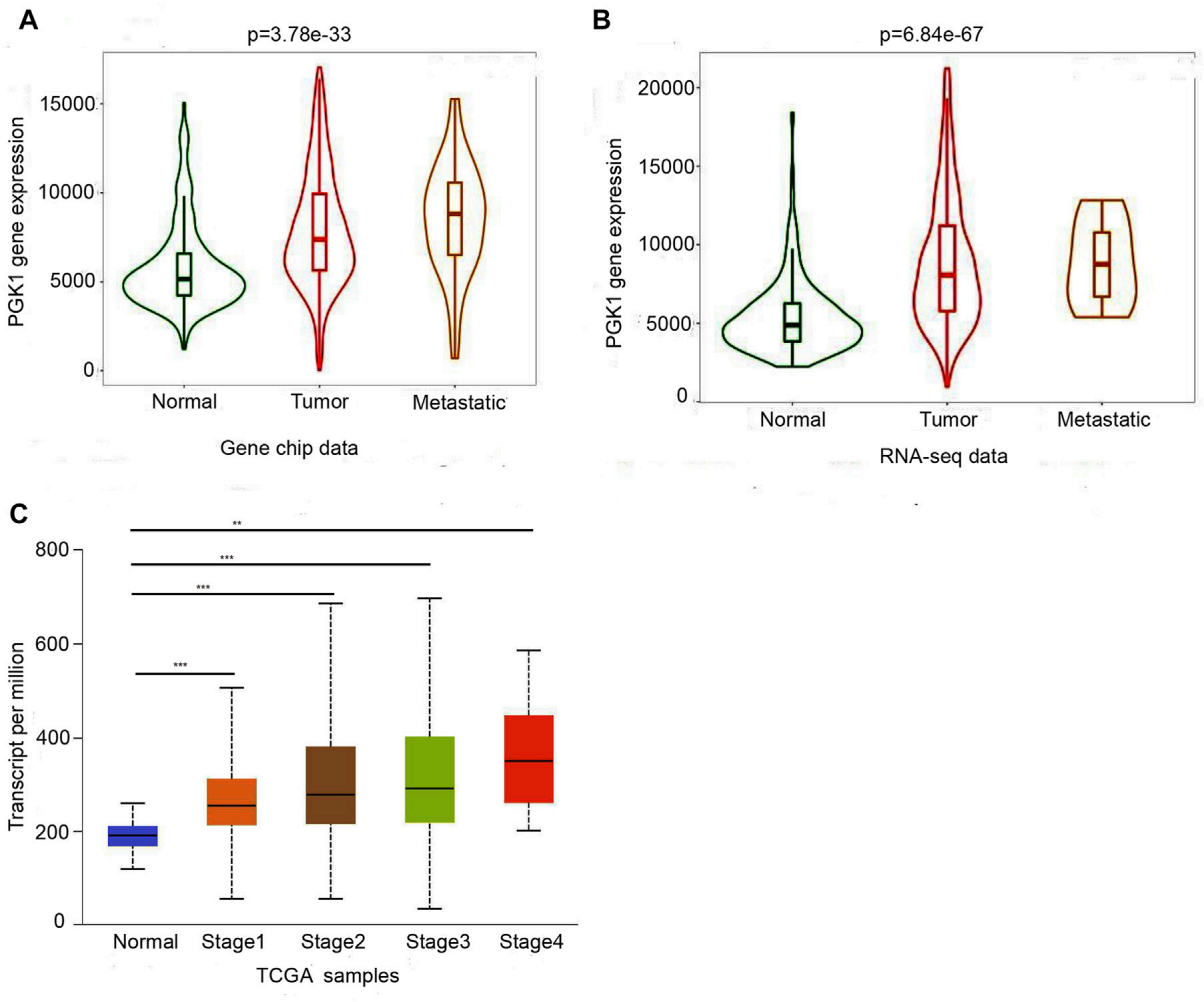
Two boxplot graphs **(A,B)** of PGK1 gene expression in breast cancer by comparing paired normal, tumor, and metastatic tissues from gene chip data and RNA-seq data at TNMplot.com. Validation of differential expression using equally sized training and test sets confirmed the reliability of the database in breast cancer at an FDR below 10%.**(C)**Relationship between PGK1 expression and tumor stage in breast cancer patients. Boxplots displayed the PGK1 expression between normal and breast cance patients with stage 1, 2, 3, and 4, * means *p* < 0.05; ** means *p* < 0.01; ***means *p* < 0.001.

Moreover, the PGK1 expression level was apparently elevated in different subtypes of BRCA as well, which include invasive ductal breast carcinoma (*p* = 1.20 × 10^−32^) (Table 1 and Figure 2A), intraductal cribriform breast adenocarcinoma(*p* = 8.98 × 10^−4^) (Table 1 and Figure 2B), invasive ductal and lobular carcinoma(*p* = 8.52 × 10^−4^) (Table 1 and Figure 2C), mixed ductal and lobular breast carcinoma (*p* = 4.19 × 10^−4^) (Table 1 and Figure 2D), invasive breast carcinoma (*p* = 1.66 × 10^−15^) and invasive lobular breast carcinoma (*p* = 9.32 × 10^−8^) (Table 1 and Figure 2E,F), compared with the normal tissue. Analysis PGK1 expression using the TNM plot analysis showed that PGK1 expression is higher in metastatic tissues than normal and tumor tissues from gene chip data and RNA-seq data (Table 2 and Figures 3A,B) (*p* = 3.78e–33, *p* = 6.84e–67). The mRNA expressions of PGK1 are also remarkably correlated with the cancer stage, and patients with advanced cancer stages tended to express higher mRNA expression of PGK1 (Figure 3C). Together, these results indicated that PGK1 mRNA expression was up-regulated in human breast cancer.

**TABLE 1:** Significant changes in PGK1expression at the transcription level between different types of breast cancer and normal tissues (oncomine database).

Subtype of breast cancer	*p*-value	Fold-change	Rank (%)	Sample
Invasive ductal breast carcinoma	1.2 × 10^−32^	2.003	3	450
Intraductal cribriform breast adenocarcinoma	8.89 × 10^−4^	2.109	6	64
Invasive ductal and lobular carcinoma	8.52 × 10^−4^	1.397	7	64
Mixed lobular and ductal breast carcinoma	4.19 × 10^−4^	1.585	7	68
Invasive breast carcinoma	1.66 × 10^−15^	1.621	6	137
Invasive lobular breast carcinoma	9.32 × 10^−8^	1.507	11	97

**TABLE 2:** Compare expression profiles of tumor, normal and metastatic tissues.

Data	n.Norm	n.Tumor	n.Meta	K.W.p	Fc.tumor.norm	Fc.tumor.norm
Gene chip data	242	7,659	82	3.78e–33	1.42	1.05
RNA-seq data	403	1,097	7	6.84e–67	1.84	0.89

### PGK1 Protein was High-Expressed in BRCA Patients

**FIGURE 4:**
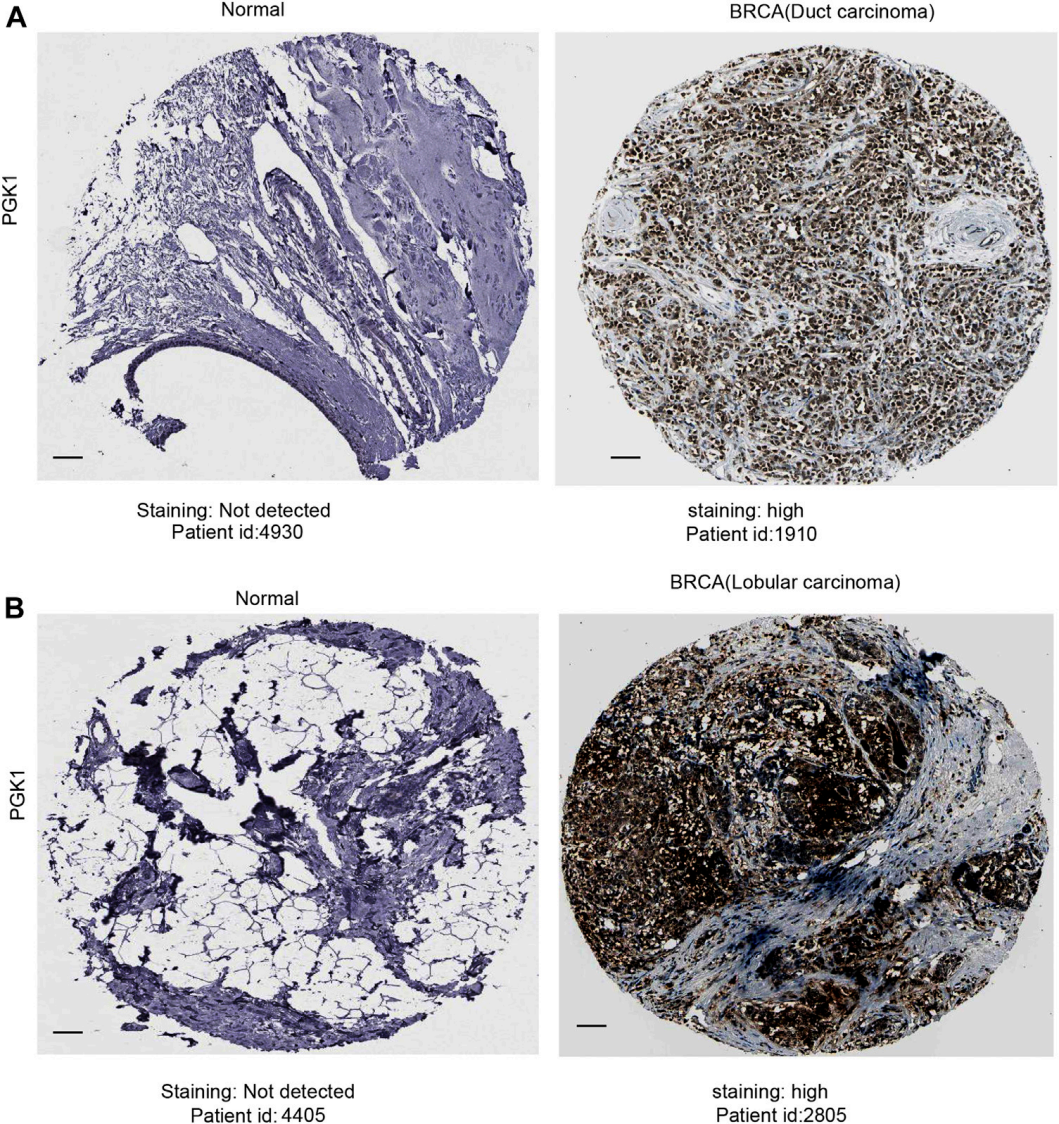
Representative immunohistochemistry images of PGK1 in duct carcinoma **(A)** and lobular carcinoma (**B**) tissues and normal breast tissues (HPA database) were shown. Scale bar: 50 μM.

After analyzing the mRNA expression of PGK1 in BRCA, we explored the protein expression of PGK1 using the Human Protein Atlas (HPA). PGK1 protein has high expressions in BRCA tissues (Ductal carcinoma and Lobular carcinoma), while it has low protein expression in normal breast tissues by HPA (Figures 4A,B). The above results showed that protein expression of PGK1 was high-expressed in patients with breast cancer.

### The Correlation of PGK1 with the Breast Cancer Clinicopathologic Parameters

**FIGURE 5:**
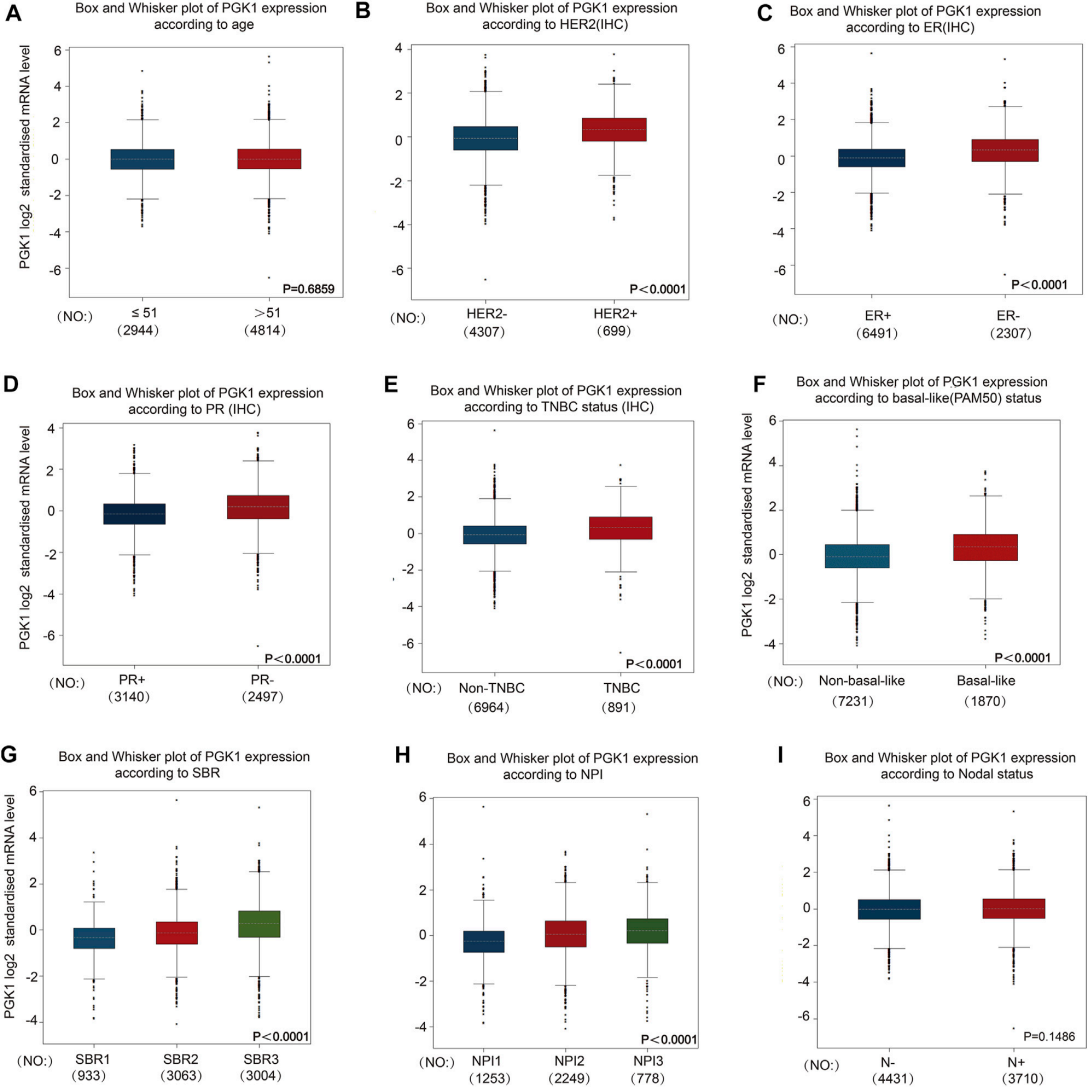
To study the correlation of PGK1 expression with clinical pathological parameters including age **(A)**, HER2 **(B)**, ER **(C)**, PR **(D)**, TNBC status **(E)**, basal-like (PAM50) status **(F)**, SBR **(G)**, NPI **(H)** and Nodal status **(I)** in breast cancer patients. Notable differences between the groups were analyzed by Welch’s *t*-test to generate the *p*-value. PGK1, phosphoglycerate kinase 1; ER, estrogen receptor; PR, progesterone receptor; HER2, human epidermal growth factor receptor 2; SBR, Scarff, Bloom and Richardson; TNBC, Triple-negative Breast Cancer; NPI, Nottingham Prognostic Index status; IHC, immunohistochemistry.

To evaluate the correlation of PGK1 expression with the clinicopathologic parameters of BRCA, we performed the analysis by bc-GenExMiner v4.5. The age criterion demonstrated there is no difference of PGK1 mRNA expression in patients tumors of aged over 51 (year) vs. the aged no more than 51 (*p* > 0.5) (Figure 5A and Table 3). Additionally, PGK1 mRNA expression was obviously up-regulated in estrogen receptor (HER2) (+) group vs. the corresponding HER2 (−) group (*p* < 0.0001) (Figure 5B and Table 3). Moreover, the PGK1 mRNA expression was apparently reduced in progesterone receptor (PR) (−) group vs. the PR (+) (*p* < 0.0001) (Figure 5C and Table 3). Similarly, PGK1 mRNA expression was also reduced estrogen receptor (ER) (−) group vs. the ER (+) group (*p* < 0.0001), compared with the corresponding negative group (Figure 5D and Table 3). Furthermore, the expression of PGK1 was determined in Triple-negative breast cancer (TNBC) and found that PGK1 mRNA expression was significantly up-regulated in TNBC patients (*p* < 0.0001) (Figure 5E and Table 3). Consistently, patients with basal-like characteristics also exhibited apparently increased PGK1 expression vs. patients with non-basal-like characteristics (*p* < 0.0001) (Figure 5F; Table 3). In the Nottingham Prognostic Index (NPI) criterion and Scarff Bloom and Richardson grade status (SBR), an increased NPI and SBR grade was correlated with PGK1 transcript level increase (*p* < 0.0001) vs. the SBR1 and NPI1 groups correspondingly (Figures 5G,H). However, there was no significant difference in terms of human nodal status (Figure 5I and Table 3). Taken together, the above results showed a prognostic value in breast cancer clinicopathologic parameters.

**TABLE 3:** The correlation between PGK1 mRNA expression and the clinicopathological parameters of breast carcinoma.

Variables	PGK1		
	Number	mRNA	*p*-value
Age,years			0.6859
≤51	2,944	−	
>51	4,814	−	
HER2			<0.0001
−	4,307	−	
+	699	↑	
ER			<0.0001
−	2,307	↑	
+	6,491	−	
PR			<0.0001
−	2,497	↑	
+	3,140	−	
Triple-negative status			<0.0001
None	6,964	−	
TNBC	891	↑	
Basal-like status			<0.0001
None	7,231	−	
Basal-like	1870	↑	
Nodal status			0.1486
−	4,431	−	
+	3,710	−	

### The Correlation of PGK1 Expression with Crucial Genes Mutations

**FIGURE 6:**
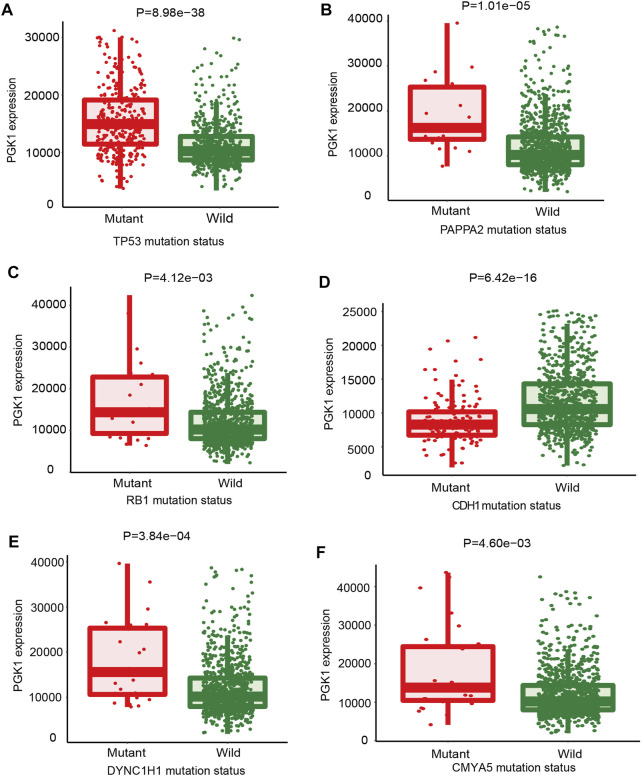
The mutations that correlated with PGK1 gene expression in breast cancer were identified. The oncogenes or tumor suppressor genes mutations correlated with PGK1 expression was analyzed by TARGET from muTarget platform. The six genes whose mutations are most strongly correlated with PGK1 expression changes in breast cancer. TP53, tumor protein p53 **(A)**; PAPPA2, pappalysin 2 **(B)**; RB1, RB transcriptional co-repressor 1 **(C)**; CDH1, cadherin 1 **(D)**; dynein cytoplasmic 1 heavy chain 1 **(E)**; CMYA5, cardiomyopathy associated 5 **(F)**. Screening of significant genes were based on the Mann-Whitney *p* value and mean FC of the test, *p* ≤ 0.01 and 0.714>FC>1.4 and a prevalence of at least 1%.

In order to identify the correlation of mutations which could guide the therapy of breast cancer and PGK1 expression, the Mann-Whitney U analysis was performed to identify crucial gene mutations correlated with PGK1 expression. The condition of selected genes was FC > 1.4, *p* < 0.01. As shown in Figure 6, the six most strongly associated genes with PGK1 expression genes were described. PGK1 expression was higher in TP53-mutant (total mutation rate: 34%), PAPPA2-mutant (total mutation rate: 2%), RB1-mutant (total mutation rate: 2%), DYNC1H1-mutant (total mutation rate: 2%), and CMYA5-mutant (total mutation rate: 2.7%) breast cancer patients (Figures 6A–C,E,F). PGK1 expression was lower in tumor specimens containing somatic mutations of CDH1 (mutation prevalence 14.1%) (Figure 6D). The list of six genes with PGK1 expression change associated somatic mutations is presented in Table 4. Collectively, these results suggested that PGK1 expression has a closely correlation with gene mutations in breast cancer.

**TABLE 4:** The six mutant genes most strongly associated with PGK1 expression.

Mutation of genes	Mean expression (mutant)	Mean expression (wild)	Number of mutant	Number of wild	FC (mutant/wild)	Direction	*p*-value
TP53	15,821.95	10,607.23	336	643	1.49	Up	8.98e–38
PAPPA2	19,023.6	12,258.76	20	959	1.55	Up	1.01e–05
RB1	18,605.74	12,247.59	23	956	1.52	Up	4.12e–03
CDH1	8,842.55	12,980.2	138	841	1.47	Down	6.42e–16
DYNC1H1	17,994.04	12,256.3 24	24	955	1.47	Up	3.84e–04
CMYA5	17,682.93	12,247.02	27	952	1.44	Up	4.60e–03

### Construction Gene Interaction Network and Function Enrichment of PGK1 in Breast Cancer

**FIGURE 7:**
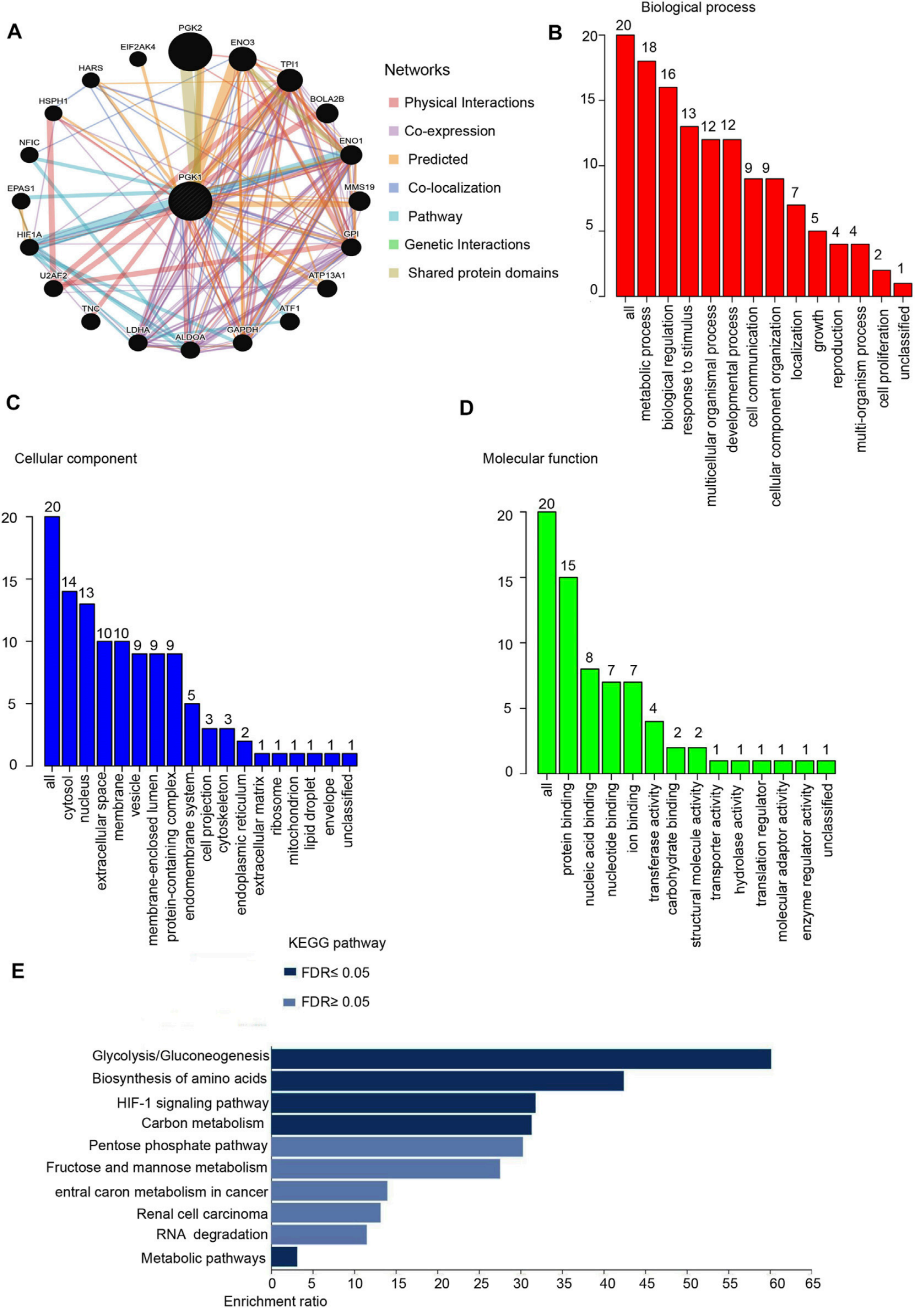
Protein-protein interaction network and function enrichment analysis of PGK1 in breast cancer. **(A)** Protein-protein interaction network of PGK1 was analyzed by GeneMANIA and Screening for 20 genes interacting with PGK. Different colors of the network edge indicate the bioinformatics methods applied: Physical Interactions, Co-expression, Predicted, Co-localization, Pathway, Genetic Interactions, Shared protein domains. **(B)** Gene Ontology (GO) is analyzed and the screened 20 gene of BP (Biological Process) is shown. **(C)** CC (Cellular Component). **(D)** MF (Molecular Function). **(E)** KEGG pathways is analyzed the 20 genes co-expressed with PGK1 and the Top pathway is mapped according to the differential expression level of PGK1 (*p* ≤ 0.05). *Y*-axis: name of the signaling pathway or function; *X*-axis: percentage of the number of genes assigned to a term among the total number of genes annotated in the network.

In this study, we constructed a network for PGK1 and their 20 related genes and analyzed their functions using GeneMANIA. Proteins that interact with PGK1 are ATF1, GPI, TPI1, ATP13A1, EPAS1, TNC, NO1, LDHA, HSPH, EIF2AK4, ENO3, HIF1A, BOLA2B, U2AF2, NFIC, MMS19, PGK2, HARS, ALDOA, GAPDH (Figure 7A). Moreover, we performed the analysis of the physiological functions and biological processes of the 20 genes by using the Web Gestalt, the results showed that 20 genes were classified into the following categories: molecular function, cellular component, and biological process. Notably, most genes are enriched in the metabolic process, biological process, and protein binding et al. (Figures 7B–D). In addition, the Go and pathway functional analysis revealed that these proteins showed the greatest correlation with metabolic processes. These genes were significantly enriched in glycolysis/gluconeogenesis, biosynthesis of amino acids, HIF-signaling pathway, and carbon metabolism (Figure 7E and Table 5). The above results showed construction gene interaction network and function enrichment of PGK1 in breast cancer.

**TABLE 5:** KEGG Pathway enrichment analysis of PGK1 gene–gene interaction network.

Gene set	FDR	*p*-value	Enrichment ratio	Overlap genes
hsa00010:Glycolysis/Gluconeogenesis	1.1220e–12	3.4417e–15	60.146	ALDOA,ENO1ENO3, TPI1 GAPDH,GPI,PGK1,PGK2,LDHA
hsa01230:Biosynthesis of amino acids	1.0780e–8	9.9204e–11	42.414	ALDOA,ENO1ENO3,TPI1, PGK2 GAPDH,PGK1
hsa04066:HIF-1signaling pathway	6.3447e–8	7.7849e–10	31.811	ALDOA,ENO1ENO3,HIF1A,GAPDH,PGK1,LDHA
hsa01200:Carbon metabolism	6.2082e–9	3.8087e–11	31.341	ALDOA,ENO1ENO3,TPI1, PGK2 GAPDH,PGK1,GPI
hsa00030:Pentose hosphate pathway	0.10352	0.0019053	30.296	ALDOA, GPI
hsa00051: Fructose and mannose metabolism	0.10729	0.0023037	27.542	ALDOA, TPI1
hsa05230:Central carbon metabolism in cancer	0.35406	0.0087108	13.983	HIF1A, LDHA
hsa05211:Renalcell carcinoma	0.35406	0.0097746	13.172	EPAS1, HIF1A
hsa03018:RNA degradation	0.41317	0.012674	11.505	ENO1ENO3
hsa01100:Metabolic pathways	0.041688	0.00063938	3.1341	ALDOA,ENO1ENO3, TPI1 GAPDH,GPI,PGK1,PGK2,LDHA

### PGK1 Acts as an Independent Indicator for BRCA Prognosis

**FIGURE 8:**
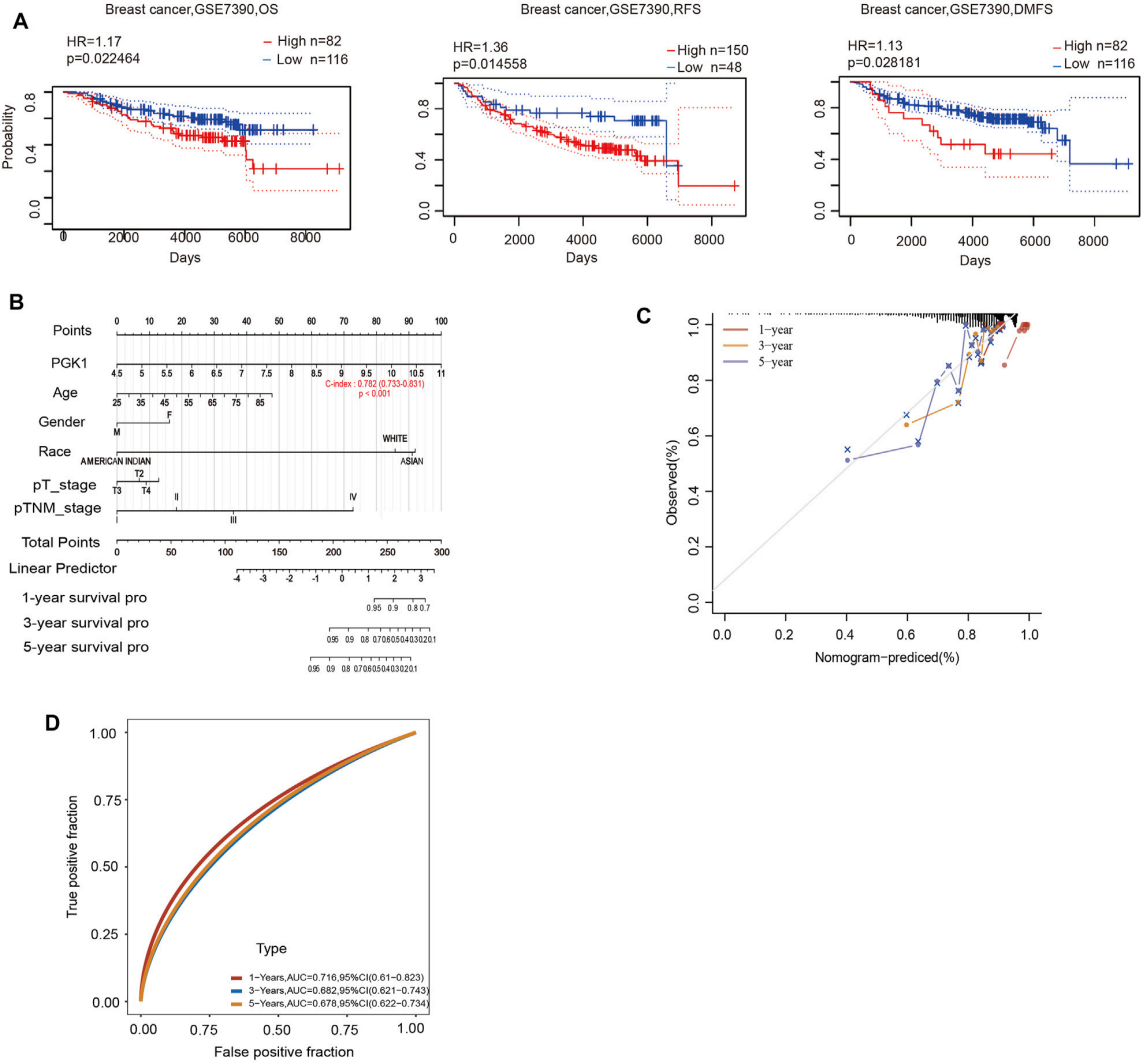
**(A)** Prognostic significance of PGK1gene expression in patients with breast cancer (OS, RFS and DMFS time in the PrognoScan database). OS, overall survival; RFS, relapse-free survival; DMFS, distant metastasis free survival; HR, hazard ratio. **(B)** Nomogram to predict the 1-year, 3-years, and 5-years overall survival of breast cancer patients. **(C)** Calibration curve for the overall survival nomogram model in the breast cancer group.A dashed diagonal line represents the ideal nomogram, and the red line, orange line, and blue line represent the 1-year,3-years, and 5-years observed nomograms. **(D)** Time-dependent ROC analysis the of the five-gene signature. ROC, receiver operating characteristic.

To determine the correlation of PGK1 expression with breast cancer patients survival, we performed the analysis and showed that increased expression of PGK1 mRNA is apparently correlated with reduced overall survival (OS) (*p* < 0.05), relapse free survival (RFS) (*p* < 0.05), and distant metastasis free survival (DMFS) in breast cancer (*p* < 0.05) (Figure 8A). Cox analysis was performed to assess the independent prognostic value of PGK1, and the results are shown in Table 6 and Table 7. According to the results from univariate and multivariate analysis, PGK1 gene, age, and pTNM stage were significantly associated with OS. The above results indicated that the PGK1 gene, age, and pTNM stage were independent prognostic factors in breast cancer. Based on multivariate Cox Regression analysis, variables with significant prognostic differences were automatically extracted using the tool (www.aclbi.com) to construct a prognostic nomogram for BRCA patients (Figure 8B). Moreover, as shown in Figure 8C, the calibration analysis results showed that the nomogram for 1-year survival rate (the red line) was highly approached to the ideal performance (the 45-degree gray line) vs. to the 3-years (the orange line) and 5-years (the blue line) survival rates, which suggested a remarkably accurate nomogram predicted by this model. The AUC can observe the efficacy of the gene as a prognostic biomarker, so AUC curves were obtained using R package. The result shows that AUC values at 1, 3, and 5 years are 0.716 (95% CI, 0.61−0.823), 0.682 (95% CI, 0.621−0.743), and 0.678 (95% CI, 0.622−0.734), respectively. The above results indicated the strong predictive ability of PGK1 for breast cancer (Figure 8D).

**TABLE 6:** Hazard ratio and *p*-value of constituents involved in univariate Cox regression and some parameters of the PGK1 gene.

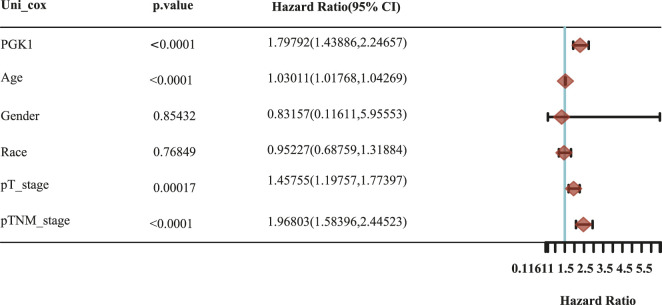

**TABLE 7:** Hazard ratio and *p*-value of constituents involved in multivariate Cox regression and some parameters of the PGK1 gene.

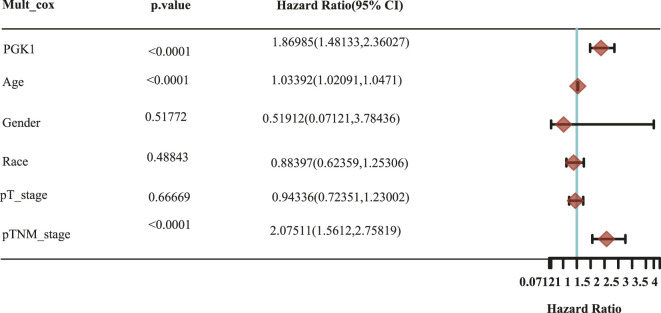

## Discussion

Along with the rapid development of precision medicine, accurate prognostic models are needed to guide clinical and to design a more personalized program for patients, especially those with a complex type of breast cancer. Moreover, breast cancer cells are easy to metastasize and are life-threatening (Yang et al., 2019). Aside from the role of PGK1 as a glycolytic enzyme in different cell cycle intervals, PGK1 also shows an indispensible role in tumor metabolism. The Aberrant expression of PGK1 in various tumor tissues, peripheral blood and saliva of patients, could promote rapid tumor growth and progression (Yu and Li, 2017). Abnormal expression level of PGK1 was detected not only in tumor tissues, but also in patients’ peripheral blood and saliva. Therefore, PGK1 is a potential target of tumor therapy and an intensely studied molecule in tumor therapy research (Hwang et al., 2006). PGK1 promotes the breast cancer cell growth and the lactic acid generation, which is the end product of glycolysis (Li et al., 2016). In addition, PGK1 high expression was also representing higher tumor stage, which disclosed that PGK1 was correlated with tumor metastasis, progression and occurrence of breast cancer (Fu et al., 2018). However, the expression patterns of PGK1 in various types of breast cancer and the unique roles of PGK1 as a diagnostic marker of poor prognosis in breast cancer remain unknown.

In the previous studies, Sun et al. have proved that PGK1 expression was increased in breast cancer tissues vs. the normal breast tissues (Sun et al., 2015). Based on the existing research, we synthesized and analyzed multiple data sources including the Oncomine, UALCAN, TNMplot, and HPA database, the results showed that the PGK1 mRNA expression was also significantly upregulated in invasive ductal breast carcinoma, intraductal cribriform breast adenocarcinoma, invasive ductal and lobular carcinoma, mixed lobular and ductal breast carcinoma, invasive breast carcinoma, and invasive lobular breast carcinoma using the Oncomine database in-depth analysis. Following the TNMplot and UALCAN database data showed that the PGK1 transcriptional level was remarkably correlated with metastasis and cancer stages. Moreover, we found that the protein expression of PGK1 was higher in breast cancer tissues than in normal tissues by HPA. In addition, bc-GenExMiner 4.5 was used to investigate the expression profile of PGK1 across PAM50 breast cancer subtypes based on different clinicopathological parameters. High expression of PGK1 was associated with risk of HER (+), TNBC, basal-like characteristics, SBR grade status, and NPI grade status. However, the PGK1 mRNA expression was significantly downregulated in patients with ER (+) and PR (+) status.

HIF-1α serves as a transcriptional factor to promote cell glycolysis for Warburg effect. As an essential enzyme in the glycolytic pathway, it is proved that PGK1 is directly regulated by HIF-1α in many cancer types (Xie et al., 2017; Sun et al., 2019). Furthermore, PGK1 is relative with cancer cell metastatic ability because HIF-1α/PGK1 mediated epithelial-mesenchymal transition (EMT) process (Ai et al., 2011). In our analysis of PGK1 correlated signaling pathways in breast cancer, GeneMANIA and Web Gestalt analysis indicates PGK1 is involved in glycolysis/gluconeogenesis, biosynthesis of amino acids, HIF-signaling pathway, carbon metabolism and metabolic pathways. The results are consistent with previous studies and also indicate PGK1 acts energetically role in tumor cell metabolism. However, more details of PGK1 mediated tumor metabolism related signaling pathways need to be further investigated in future researches.

Accurate prognostic models can be used to guide the clinical decision-making process and design more personalized treatment plans for patients (Cao et al., 2019). Existing data analysis showed that elevated expression of PGK1 was related with short OS (Overall Survival) in diverse types of cancer (Shao et al., 2019) or PGK1 combined with other genes to predicting the survival of patients with breast cancer (Zhang et al., 2021). However, the performance of existing prognostic models is limited. Here, we in-depth analyzed the prognostic impact of PGK1 and clinical factors such as age, gender, race, and pTNM stage. First, the survival results revealed that high expression of PGK1 mRNA was associated with reduced OS, RFS, and DMFS via PrognoScan analysis. PGK1 was found to be an independent prognostic factor in determining breast cancer by univariate and multivariate cox regression. The PGK1-based model of breast cancer showed moderate predictive ability (AUC = 0.716, 0.682, and 0.678 for 1, 3, and 5-years survival). Thus, the PGK1 signature was an independent and the most important risk factor of the prognosis of breast cancer.

In addition to single gene indicators, multigene prognostic models have also gained acceptance in recent years (Győrffy et al., 2015). However, large groups of breast cancer patients harboring different somatic mutations still do not have adequate targeted therapies. Thus, we used the muTarget software to identify mutations that alter the expression of the PGK1 gene. The TP53 gene encodes a tumor suppressor protein, and TP53 mutations influence the prognosis significantly in most cancer patients (Olivier et al., 2010; Li et al., 2020). Breast cancer accounts for over 50% of tumors in TP53 mutation carriers (Silwal-Pandit et al., 2017; Schon and Tischkowitz, 2018). Notably, we showed that PGK1 expression was higher in TP53-mutant than wild-type in breast cancer patients by muTarget analysis. CDH1 is another commonly mutated gene in breast cancer (Padmanaban et al., 2019). It encodes a tumor suppressor protein and its mutation contributes to the promotion of breast cancer invasion and metastasis (Lei et al., 2002). The current analysis showed that PGK1 expression was lower in CDH-mutant than wild-type in breast cancer patients. By linking TP53 and CDH1 mutations and PGK1 expression, it is possible to identify potential multi-gene therapeutic targets and develop novel personalized therapies in breast cancer.

## Conclusion

In conclusion, our results indicate the overexpression of PGK1 at the transcriptional and protein levels in breast cancer patients. Moreover, PGK1 was significantly associated with different molecular types, metastasis status, and patient’s pTNM stage. Furthermore, high expressions of PGK1 were significantly related with shorter OS, RFS, and DMFS in breast cancer patients. Notably, PGK1 could be used as an independent prognostic marker in breast cancer patients. In summary, PGK1 could be a potential target in the development of anti-PGK1 therapeutics and an efficient marker for the prognosis of breast cancer. The present study was hypothetically driven and performed using experimental generated data available in public databases. Therefore, future experimental verification of the mechanism underlying PGK1 regulation of tumor metastasis and its multi-gene prognostic value for breast cancer patients is warranted.

## Data Availability

The datasets presented in this study can be found in online repositories. The names of the repository/repositories and accession number(s) can be found in the article/Supplementary Material.
